# Degree of Compliance of Hospital Emergency Departments With the Recommended Standards and Their Evolution During the SARS-CoV-2 Pandemic

**DOI:** 10.1155/jonm/4228788

**Published:** 2025-06-25

**Authors:** Cristina Font-Cabrera, Jordi Adamuz, Maria Eulàlia Juvé-Udina, Miquel Sánchez, Almudena Mateos-Dávila, José Antonio Sarria-Guerrero, Andrea Pastor-Puigdomènech, Eva Maria Guix-Comellas

**Affiliations:** ^1^Department of Fundamental and Clinical Nursing, Faculty of Nursing, University of Barcelona, Barcelona, Catalonia, Spain; ^2^Emergency Department, Bellvitge University Hospital, Barcelona, Catalonia, Spain; ^3^Bellvitge Institute of Biomedical Research, IDIBELL Nursing Research Group, Barcelona, Catalonia, Spain; ^4^Nursing Knowledge Management and Information Systems Department, Bellvitge University Hospital, Barcelona, Catalonia, Spain; ^5^Catalan Institute of Health (ICS), Barcelona, Catalonia, Spain; ^6^Emergency Department, Clínic University Hospital, Barcelona, Catalonia, Spain

**Keywords:** emergency department, hospital, long lengths of stay, nursing, triage, waiting times

## Abstract

**Aim:** To analyse the degree of compliance of the care times of hospital emergency departments (EDs) in Spain with the recommended standards and their evolution during the SARS-CoV-2 pandemic.

**Design and Methods:** An observational, correlational, cross-sectional and retrospective study was carried out. All adult patients attended in the EDs of 8 Spanish public hospitals from 2018 to 2021 were consecutively included. The main variables were time spent in the ED, time to triage, waiting time until the start of care, triage level, classified according to the Spanish Triage System and year of care. Other sociodemographic variables were collected, in addition to discharge destination. For each triage level, a negative binomial regression model was performed, adjusted for year, hospital and waiting times. The analysis was performed with *R* 4.2.2 software.

**Results:** A total of 2,282,555 patients were included; ED lengths of stay varied according to triage levels: Level 1, 21.6 h; Level 2, 26.3 h; Level 3, 22.2 h; Level 4, 8.1 h and Level 5, 10.3 h. Statistically significant differences were detected only in 2021, in all hospitals and from priority Level 2–5. An increase in dwell times was observed in all hospitals, with longer dwell times in high complexity hospitals. Longer waiting times at triage Levels 3, 4 and 5 presented a higher risk of mortality. The percentage of patients leaving the ED was high (3.6%).

**Conclusions:** The degree of compliance with ED care times according to recommended standards was low. The dropout rate, waiting times for initial triage and ED attendance were higher than desired. The SARS-CoV-2 pandemic changed the pattern of ED visits, decreasing the frequentation of patients, but did not decrease the length of stay in the ED. This pattern normalised the following year.

## 1. Introduction

Hospital emergency departments (EDs) are one of the main gateways to the healthcare system and play a critical role, as they are often unable to guarantee an efficient and quality response to citizens. It is of concern that the majority of ED visits are considered nonurgent or inappropriate, classified as low complexity in different countries around the world [1, 2].

During the 1960s, with a marked increase in the number of patients in the ED, the need arose to distinguish between critically ill and less critically ill patients [3]. This situation prompted the global implementation of ED triage. Triage consists of a classification system that prioritises patients according to the severity of their illnesses or injuries, thus allowing more effective healthcare intervention in the shortest possible time [4, 5]. There are several validated scales for the classification of patients in the ED, such as the National Triage Scale for Australasian Emergency; the Canadian Emergency Department Triage Acuity Scale; the Manchester Triage System; the Severity Index and the Andorran Triage Model (MAT), which was adapted to the Spanish context as the Spanish Triage System (SET). This adaptation was developed by the Spanish Society of Emergency Medicine (Sociedad Española de Medicina de Urgencias y Emergencias) [6–8].

Triage is considered essential in ED management and is used as an indicator in the evaluation and validation of services [9]. Its main objective is to prioritise patients according to severity, and its precision and application in the first minutes of admission to the ED are crucial to avoid possible negative consequences for the patient's health [5]. In addition, waiting times are considered indicators of quality of care, and are defined as the time between arrival at the ED and care by the healthcare professional [10]. Long waiting periods and prolonged stay of patients in the ED are associated with increased morbidity and mortality, as they delay the start of appropriate treatment. This reduces the quality of care, leading to patient dissatisfaction and stress [11–13].

Therefore, the aim of the present study was to analyse the degree of compliance of Spanish hospital EDs with the recommended standards and their evolution during the SARS-CoV-2 pandemic.

## 2. Design and Methods

An observational, correlational, cross-sectional and retrospective study was conducted in the 8 public hospitals in Spain attached to the Catalan Health Institute (ICS). The ICS stands out as the country's leading public health institution, providing health services to almost six million people. At the hospital level, it manages 8 key hospitals within the public network. Its financing comes from the public funds of the National Health System, obtained through public taxes. Its care structure is organised into three large hospitals with advanced technology and five regional hospitals. This configuration makes it possible to effectively address healthcare needs, from the most complex to the simplest cases, providing comprehensive medical coverage for the population.

We consecutively included all users who attended the ED for a health problem from 2018 to 2021 (both included), attending a total of 2,572,365 visits. Patients under 18 years of age and women with obstetric pathology were excluded.

**Figure 1:**
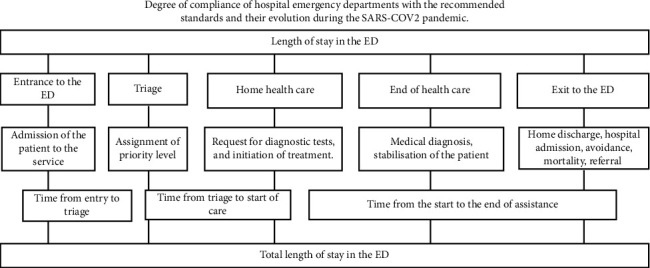
Timing diagram in the study.

Health problems were grouped according to the MAT/SET programme into five categories of being: priority 1: resuscitation, priority 2: emergency, priority 3: urgency, priority 4: less urgent and priority 5: nonurgent [14]. For secondary variables, the eight ICS hospitals were considered, the first three being considered high complexity due to their accreditation to carry out more complex care procedures. The remaining hospitals are considered low complexity. The high complexity hospitals were identified as HT1, HT2 and HT3, while the low complexity hospitals were LT1, LT2, LT3, LT4 and LT5. In Spain, hospitals are classified into HT and LT hospitals according to their health resources, equipment and available services. HT hospitals offer basic services but are mainly characterised by providing specialised and complex treatments to patients. On the other hand, LT hospitals offer more basic health care. The different years 2018, 2019, 2020 and 2021 were taken into account. Several variables related to ED waiting times were analysed. The length of stay was considered from patient admission to the ED, including the admission process, triage, healthcare until discharge or hospital admission. In addition, other waiting times were evaluated, such as triage time, which was measured from the patient's arrival at the ED until the nursing staff carried out the initial assessment (triage). During this process, the nurse determined the patient's priority level according to the severity of the patient's situation. The start and end of care time was also analysed. The start of care was defined as the time from triage to the start of care. This changes according to the priority level assigned. Priority 1 requires immediate attention from healthcare staff; Priority 2 must be seen in less than 15 min; Priority 3 must not exceed 60 min waiting time; Priority 4 has a maximum waiting time of 120 min and Priority 5 can wait up to 240 min for care [2]. Finally, end of care is considered the time at which the patient is discharged, admitted, referred, evaded or exitus (Figure 1). Data were extracted retrospectively from the ED Minimum Basic Data Set (MBDS) registry.

In structured triage, operational objectives have been established to measure and guarantee the efficiency and quality of the process. In Spain, the Spanish Society of Emergency Medicine (SEMES) highlights triage as a primary indicator of quality of care. It establishes key performance indicators that are not standards but ideal goals: (a) ensure that ≤ 2% of patients leave without receiving medical attention after registration; (b) initiate triage within ≤ 10 min of arrival at the ED and (c) ensure that at least 90% receive care within 2 h of triage, reaching 100% within 4 h [15].

Categorical variables were described by the number of cases, the percentage of the total by category and the number of missing data. Continuous variables following a normal distribution were described by the number of cases, the mean, the standard deviation and the number of missing data. Continuous variables that did not follow a normal distribution were described by the number of cases, the median, the first and third quartiles and the number of missing data. Median times for each triage level across years and by hospital were plotted. Quantile regression models were performed for the 90th percentile, adjusting by year and hospital. Percentile estimators were presented together with the corresponding 95% confidence interval. The analysis was performed with *R* 4.2.2 software.

The study was carried out in accordance with the regulations and laws in force in our country, through the Organic Law on Data Protection and Guarantee of Digital Rights. In order to guarantee patient confidentiality, an anonymisation system managed by the technical secretariat of the ICS corporate centre was set up, removing all data that could be used to identify them. The research had the support of the Research Ethics Committee of the Bellvitge University Hospital under the code (PR085/20).

## 3. Results

**Figure 2:**
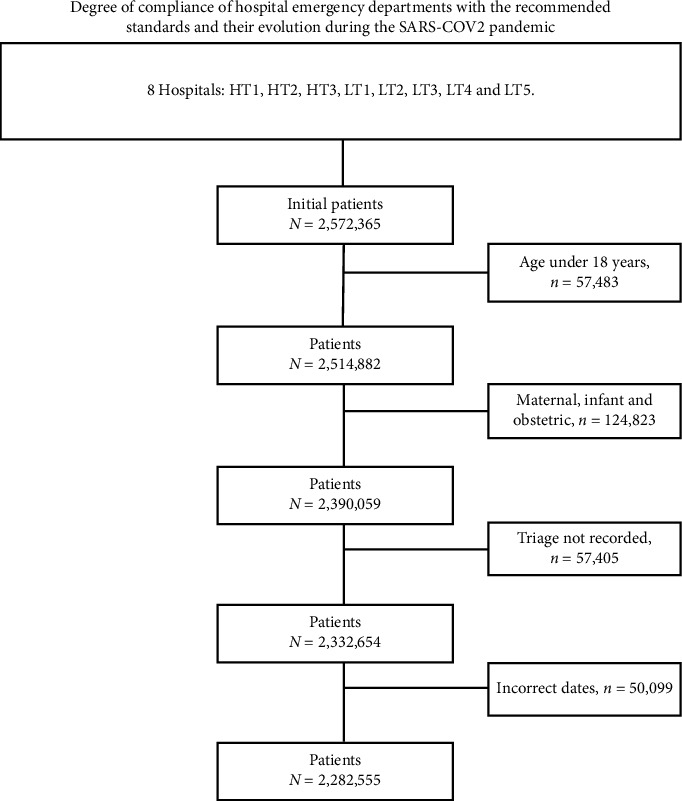
Flowchart for patient enrolment in the study.

**Figure 3:**
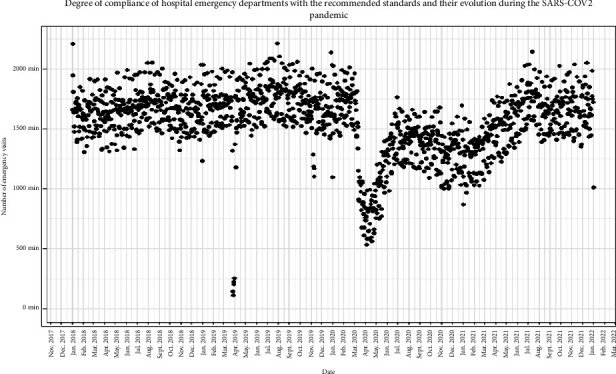
Total number of visits by year.

A total of 2,572,365 patients were seen. Of these, 2,282,555 patients met the study inclusion criteria (Figure 2). A total of 51.4% (*n* = 1,173,233) were women, and the mean age was 55.4 years (SD = 20.7). By year, there were 603,897 visits in 2018, 618,766 in 2019, 48,728 in 2020, where it was observed that the SARS-CoV-2 pandemic caused the number of ED visits to decrease markedly and 572,608 in 2021, where there was a recovery of the pattern of ED patient visits to levels similar to the prepandemic period (Figure 3). Length of stay in the ED was notably prolonged at all triage levels. At priority level 1, 90% of the patients had a maximum length of stay of 21.6 h (mean = 5.1 h) in the ED. Similar results were obtained for priority levels 2 and 3 ([P90] = 26.3 h and 22.2 h and mean = 8.1 h and 5.9 h, respectively), while the ED stay times of patients with priority levels 4 and 5 were lower ([P90] = 8.1 h and 10.3 h and mean = 2.2 h in both) (Table 1). Length of stay in the ED was notoriously long at all triage levels. At priority level 1, 90% of the patients had a maximum length of stay of 21.6 h (mean = 5.1 h) in the ED. Similar results were obtained for priority levels 2 and 3 (90th percentile [P90] = 26.3 h and 22.2 h and mean = 8.1 h and 5.9 h, respectively), while the ED stay times of patients with priority levels 4 and 5 were lower (P90 = 8.1 h and 10.3 h and mean = 2.2 h in both) (Table 1).

### 3.1. Waiting Times to Triage and to Initiation of Care

Waiting time to triage was longer than 10 min at the 90th percentile at all priority levels although a clear trend was maintained: the higher the triage level, the shorter the waiting time (Level 1: P90 = 11 min and mean = 4 min; Level 2: P90 = 17 min and mean = 6 min; Level 3: P90 = 21 min and mean = 8 min; Level 4: P90 = 24 min and mean = 9 min and Level 5: P90 = 24 min and mean = 8 min).

Waiting time for initiation of care varied according to the priority level assigned to each patient (Level 1: P90 = 2 h and mean = 10 min; Levels 2 and 3: P90 = 3 h and mean = 21 and 35 min, respectively; and Levels 4 and 5: P90 = 2.5 h and mean = 35 and 30 min, respectively) (Table 1).

### 3.2. Length of Stay, Reasons for Discharge and Mortality

In high-complexity hospitals, longer lengths of stay were observed than that in low-complexity hospitals, at all priority levels (Table 2). Prolonged length of stay was associated with hospital admission, referral to other centres and a higher risk of mortality (Table 3).

Mortality and referrals were highest at the highest priority levels (1 and 2). It was observed that 60% of the patients categorised as Level 1 were admitted to hospital. Of these, 22% were discharged, 8.6% died and 8.5% were referred to other centres. As the severity level decreased, the frequency of discharge to home increased and the frequency of hospitalisation decreased. At triage Level 5, 81% of the patients were discharged, 7.8% were admitted to the ward, 0.07% died and 3.2% were referred to other facilities. In addition, a greater increase in patients leaving the ED was observed in the lower triage levels (4.36% for Level 4 and 6.61% for Level 5). In some of the higher severity levels, higher than recommended dropout rates were also detected (2.35% for Level 1; 1.74% for Level 2 and 2.96% for Level 3) (Table 3).

The reasons for staying in the ED differed according to the triage level. At Level 1, patients experienced long waits for critical care beds or for referral to other centres. In Levels 2, 3, 4 and 5, it was mainly due to hospital admission.

Analysing dwell time according to the different triage levels, significant differences were observed at all levels in 2021 compared with 2018. The time increased to 288 min at Level 1 (*p* < 0.001), 76 min at Level 2 (*p* < 0.001), 106 min at Level 3 (*p* < 0.001), 82 min at Level 4 (*p* < 0.001) and 163 min at Level 5 (*p* < 0.001). On the other hand, during 2020, coinciding with the pandemic, the increase to dwell time was only observed in the less urgent levels: Level 4 increased to 114 min (*p* < 0.001) and Level 5 to 134 min (*p* < 0.001).

In addition, significant differences in dwell time were detected between HT and LT hospitals, being longer in HTs. In triage 1, only one HT hospital showed a significant increase (*p* < 0.001). In triage 2 and 3, the largest increases in time were in HT centres (*p* < 0.001). In triage 4, increases were limited to only two HT hospitals (*p* < 0.001), while in triage 5, this time increased in two HT centres (*p* < 0.001) but decreased in the other hospitals (Table 4).

## 4. Discussion

The main factors influencing the length of stay in the ED in Catalonia are hospital admission and referral to other centres. In addition, length of stay is related to a higher risk of mortality. It is observed that the length of stay increases in highly complex hospitals and this is higher as the years go by. Although the number of ED visits decreased during the pandemic, the length of stay remained high due to the complexity of care for patients with COVID-19.

These data are consistent with previous studies such as that by Verma et al. [16], which found that prolonged ED stays were associated with increased in-hospital mortality, especially in elderly patients and those who spent more than 24 h in the ED. ED crowding and prolonged waiting times are a challenge in all countries around the world [17]. This overcapacity has negative consequences for patients, causing increased morbidity and mortality due to delays in care, affecting both MAT/SET categorised patients with high acuity levels and those with lower acuity levels [18, 19]. Patients categorised with higher levels of severity require rapid attention and intervention by the professional, while among patients with less urgency, waiting times are delayed, which can lead in some cases to them leaving the services without being visited as in the present study, where the average percentage of ED abandonment is 3.6%, much higher than the recommended 2% [20].

As ED length of stay increases, outcomes worsen, not only in the form of adverse patient outcomes but also with increased healthcare costs [19]. More critically ill patients tend to stay longer in the ED compared with those of lesser severity. This delay could be attributed to the fact that more critical patients often require hospital admission to critical, semicritical or ward beds, which are not always available [21, 22]. This low bed availability forces these patients to stay longer than necessary in the ED, contributing to the collapse of services [2]. It has been shown in the present study that, as the years go by, the length of stay is increasing. This increase may be attributed to various factors such as population ageing or the complexity of care [23].

Lengths of stay in the ED vary according to scientific evidence, ranging from 4 to 48 h [24–26]. There is no clear definition for all waiting times. Some authors define waiting time as the time from when the patient enters the ED door and ends when the patient is discharged or admitted to the hospital [18]. In contrast, others limit it to the time between arrival and first contact with a healthcare professional, be it a physician, advanced practice nurse or resident physician [21]. This definition is referred to as “door to triage”, which is the length of time one waits to be seen by the healthcare professional and is used to report on waiting times for patients in the ED [18]. According to the UK Department of Health, the total time from patient arrival in the ED to discharge should not exceed 4 h, known as the 4-h rule [27]. Excessive waiting times in the ED decrease patient safety and satisfaction, as well as increase mortality and the risk of admission to critical care units or other adverse reactions [28, 29].

One of the objectives of triage is that all patients are seen in less than 10 minutes (16); however, in routine clinical practice, as observed in our study, this time is often exceeded. In the study by Houston et al. [30], they found that patients waited longer than 10 min, while in the study by Hansen et al. [31], this time was 12 min or more. Kienbacher et al. [32] obtained a mean of 6 min, very similar to ours. It is crucial to highlight that in the most critically ill patients, any delay in the triage process can seriously impact their health [33]. Delays of up to 30 min in triage have been reported to negatively affect urgent interventions such as percutaneous coronary intervention [31].

One indicator of quality in EDs is the waiting time for care. It is stated that at least 90% of the patients should be seen within 2 h of triage [15]. In our study, this time was only met at priority Level 1. Patients who wait more than 2 h to be seen are more likely to leave the ED [31].

Our study has certain limitations. First, there may be a loss of information due to its retrospective design. There may have been reporting biases that may have influenced the interpretation of the study results although this may be corrected by the large volume of patients included. And finally, causal relationships between the different variables cannot be established as it is a descriptive study.

## 5. Conclusion

In Spain, EDs failed to attend patients within the recommended times, resulting in a low level of compliance with standards of care. The length of stay of patients in the ED was high as were the waiting times for triage and initiation of care, exceeding the optimal values. The patient abandonment rate in the ED was almost double the recommended rate. These times were even higher in HT hospitals. Such high patient lengths of stay in the ED were associated with higher mortality rates. The SARS-CoV-2 pandemic modified the pattern of visits, reducing the influx of patients but without reducing ED length of stay.

## Figures and Tables

**Table 1:** Percentiles of ED length of stay according to different categories of triage levels and different years.

	Triage 1					Triage 2					Triage 3					Triage 4					Triage 5				
	(ALL)	2018	2019	2020	2021	(ALL)	2018	2019	2020	2021	(ALL)	2018	2019	2020	2021	(ALL)	2018	2019	2020	2021	(ALL)	2018	2019	2020	2021
*Length of stay (minutes)*																									
10th percentile	71	54	66	76	86	126	124	123	124	130	96	92	96	94	100	43	43	44	43	44	36	39	39	34	33
50th percentile	307	242	274	325	362	487	467	469	504	511	355	338	333	366	392	136	131	138	135	142	134	128	142	130	137
90th percentile	1297	1183	1201	1253	1475	1582	1550	1576	1565	1626	1332	1306	1260	1331	1412	490	445	459	559	527	620	532	603	666	695

*Time from entry to triage (minutes)*																									
10th percentile	2	2	2	2	2	2	2	2	2	2	3	3	3	3	3	3	3	3	3	4	3	3	3	3	3
50th percentile	4	4	4	4	4	6	6	6	6	6	8	7	8	8	8	9	8	9	9	9	8	8	8	9	9
90th percentile	11	13	11	11	11	17	16	17	18	18	21	20	21	22	22	24	22	24	25	25	24	23	24	25	24

*Time from triage to start of assistance (minutes)*																									
10th percentile	0	0	0	0	0	0	1	1	1	0	2	3	3	2	0	0	2	0	0	0	0	1	0	0	0
50th percentile	10	8	11	15	7	21	20	22	23	19	35	37	40	32	32	35	39	38	31	30	30	36	35	26	22
90th percentile	121	80	133	168	97	180	148	184	221	179	185	180	198	192	172	153	154	162	147	146	152	154	166	143	137

*Time from start to end of the assistance (minutes)*																									
10th percentile	42	28	38	46	56	72	76	69	72	74	30	27	28	35	34	7	7	7	7	6	8	6	6	6	7
50th percentile	256	204	224	263	314	414	405	393	419	438	271	255	244	282	314	63	55	61	65	60	73	46	61	64	73
90th percentile	1219	1114	1087	1141	1437	1495	1474	1489	1449	1545	1232	1209	1147	1224	1324	391	338	350	459	531	443	437	507	575	625

**Table 2:** Length of stay in hospital emergency departments according to hospital complexity.

	Triage 1		Triage 2		Triage 3		Triage 4		Triage 5	
	Low complexity	High complexity	Low complexity	High complexity	Low complexity	High complexity	Low complexity	High complexity	Low complexity	High complexity
*Length of stay (minutes)*										
10th percentile	61	78	120	130	108	87	45	43	35	37
50th percentile	251	345	433	557	333	390	141	133	133	135
90th percentile	1084	1424	1300	1841	1049	1562	421	557	498	733

*Time from entry to triage (minutes)*										
10th percentile	2	2	2	2	3	3	3	4	3	4
50th percentile	4	4	6	6	7	8	7	10	7	9
90th percentile	10	12	15	19	18	24	19	27	19	27

*Time from triage to start of assistance (minutes)*										
10th percentile	0	0	0	1	1	3	0	1	0	0
50th percentile	6	15	14	33	31	39	33	36	24	34
90th percentile	51	166	99	267	153	226	153	153	135	163

*Time from start to end of the assistance (minutes)*										
10th percentile	39	45	84	65	42	23	9	7	7	6
50th percentile	228	279	386	452	261	286	66	61	63	58
90th percentile	1056	1338	1252	1734	980	1448	334	446	430	632

**Table 3:** Length of stay for each of the triage levels according to year and discharge status.

	Home				In-patient ward				Intensive care unit (ICU)				Abandonment of the ED				Death				Referral								
	2018	2019	2020	2021	2018	2019	2020	2021	2018	2019	2020	2021	2018	2019	2020	2021	2018	2019	2020	2021	2018	2019	2020	2021					
*TRIAGE 1: Length of stay (minutes)*																								
Years	*n* = 325 (19.9%)	*n* = 464 (22.1%)	*n* = 514 (22.4%)	*n* = 816 (25.4%)	*n* = 1023 (62.6%)	*n* = 1229 (58.4%)	*n* = 1279 (55.8%)	*n* = 1764 (54.9%)	*n* = 5 (0.31%)	*n* = 1 (0.05%)	*n* = 1 (0.04%)	*n* = 4 (0.12%)	*n* = 40 (2.45%)	*n* = 50 (2.38%)	*n* = 50 (2.18%)	*n* = 77 (2.40%)	*n* = 129 (7.89%)	*n* = 197 (9.36%)	*n* = 229 (9.99%)	*n* = 237 (7.37%)	*n* = 113 (6.91%)	*n* = 163 (7.75%)	*n* = 220 (9.59%)	*n* = 317 (9.86%)					
10th percentile	92	137	186	238	52	59	64	68	49	381	1590	121	26	12	12	12	93	105	110	115	130	147	159	199					
50th percentile	444	494	574	592	167	187	220	220	91	381	1590	1156	180	282	242	258	287	308	322	352	490	579	621	796					
90th percentile	1216	1241	1312	1348	1129	1099	1005	1451	2202	381	1590	2051	649	847	1158	1116	752	853	849	1014	1383	1541	1860	1981					

*TRIAGE 2: Length of stay (minutes)*																									
Years	*n* = 14,246 (43.1%)	*n* = 14,609 (45.6%)	*n* = 14,254 (44.2%)	*n* = 17,557 (44.4%)	*n* = 14,825 (44.8%)	*n* = 13,408 (41.9%)	*n* = 13,573 (42.1%)	*n* = 16,381 (41.5%)	*n* = 36 (0.11%)	*n* = 32 (0.10%)	*n* = 44 (0.14%)	*n* = 32 (0.08%)	*n* = 520 (1.57%)	*n* = 572 (1.79%)	*n* = 536 (1.66%)	*n* = 775 (1.96%)	*n* = 332 (1.00%)	*n* = 347 (1.08%)	*n* = 549 (1.70%)	*n* = 601 (1.52%)	*n* = 3099 (9.37%)	*n* = 3053 (9.53%)	*n* = 3278 (10.2%)	*n* = 4153 (10.5%)					
10th percentile	127	141	128	132	121	104	115	124	70	137	132	47	46	38	34	38	226	227	230	256	165	179	173	217					
50th percentile	396	411	414	424	575	551	607	593	311	524	636	854	230	249	248	241	586	677	758	729	568	635	744	847					
90th percentile	1022	1061	1116	1144	1958	2013	1830	1897	1597	1161	1424	1776	697	645	754	776	1532	1987	2083	2042	1486	1606	1820	1962					

*TRIAGE 3: Length of stay (minutes)*																										
Years	*n* = 169,509 (72.2%)	*n* = 176,730 (73.5%)	*n* = 135,685 (69.4%)	*n* = 167,811 (70.4%)	*n* = 47,063 (20.1%)	*n* = 44,726 (18.6%)	*n* = 41,789 (21.4%)	*n* = 47,984 (20.1%)	*n* = 485 (0.21%)	*n* = 456 (0.19%)	*n* = 260 (0.13%)	*n* = 311 (0.13%)	*n* = 7085 (3.02%)	*n* = 7702 (3.20%)	*n* = 5140 (2.63%)	*n* = 7169 (3.01%)	*n* = 402 (0.17%)	*n* = 394 (0.16%)	*n* = 601 (0.31%)	*n* = 573 (0.24%)	*n* = 10,090 (4.30%)	*n* = 10,438 (4.34%)	*n* = 12,112 (6.19%)	*n* = 14,686 (6.16%)					
10th percentile	81	86	81	87	254	283	283	314	37	28	28	23	59	53	50	56	235	244	292	313	205	216	282	322					
50th percentile	275	277	277	298	849	783	783	867	204	173	138	96	229	222	218	231	788	796	974	981	621	676	910	1026					
90th percentile	801	793	889	936	2325	1895	1895	1990	923	921	1061	1152	555	548	595	629	2092	2103	2414	2513	1511	1578	1875	2189					

*TRIAGE 4: Length of stay (minutes)*																											
Years	*n* = 237,665 (88.8%)	*n* = 242,685 (88.5%)	*n* = 178,208 (87.0%)	*n* = 202,202 (87.0%)	*n* = 12,348 (4.61%)	*n* = 12,265 (4.47%)	*n* = 13,595 (6.64%)	*n* = 11,753 (5.06%)	*n* = 529 (0.20%)	*n* = 531 (0.19%)	*n* = 461 (0.23%)	*n* = 539 (0.23%)	*n* = 11,716 (4.38%)	*n* = 13,083 (4.77%)	*n* = 7569 (3.69%)	*n* = 10,750 (4.62%)	*n* = 30 (0.01%)	*n* = 45 (0.02%)	*n* = 43 (0.02%)	*n* = 43 (0.02%)	*n* = 5283 (1.97%)	*n* = 5744 (2.09%)	*n* = 4982 (2.43%)	*n* = 7211 (3.10%)					
10th percentile	43	44	42	45	246	243	294	302	25	25	26	27	46	43	38	48	247	212	386	160	14	12	14	10					
50th percentile	123	129	122	132	869	786	778	767	93	89	78	80	185	188	175	199	882	663	955	717	188	182	333	63					
90th percentile	354	370	380	398	2493	2384	1847	1882	440	293	286	286	402	414	404	473	2228	2232	2375	1834	1143	1161	1547	1350					

*TRIAGE 5: Length of stay (minutes)*																												
Years	*n* = 54,963 (83.3%)	*n* = 56,783 (82.5%)	*n* = 41,620 (81.2%)	*n* = 45,649 (79.7%)	*n* = 4654 (7.05%)	*n* = 5014 (7.28%)	*n* = 4492 (8.76%)	*n* = 4644 (8.11%)	*n* = 401 (0.61%)	*n* = 345 (0.50%)	*n* = 281 (0.55%)	*n* = 317 (0.55%)	*n* = 4316 (6.54%)	*n* = 4714 (6.85%)	*n* = 3122 (6.09%)	*n* = 4004 (6.99%)	*n* = 42 (0.06%)	*n* = 33 (0.05%)	*n* = 56 (0.11%)	*n* = 52 (0.09%)	*n* = 1630 (2.47%)	*n* = 1946 (2.83%)	*n* = 1705 (3.33%)	*n* = 2596 (4.53%)					
10th percentile	39	40	36	37	157	147	156	165	20	13	11	13	26	21	17	16	47	154	215	94	16	13	13	11					
50th percentile	115	130	117	124	689	677	637	635	84	65	51	50	152	147	121	143	276	593	718	778	260	275	384	80					
90th percentile	372	440	438	479	2322	2323	1797	1890	220	230	167	182	376	410	425	440	1831	2407	2768	2414	1097	1247	1726	1520					

**Table 4:** Models for the 90th percentile of length of stay by year and hospital.

	Triage 1			Triage 2			Triage 3			Triage 4			Triage 5		
	Estimates	95% CI	*p* value	Estimates	95% CI	*p* value	Estimates	95% CI	*p* value	Estimates	95% CI	*p* value	Estimates	95% CI	*p* value
*Year*															
Intercept	1187	1111.57–1262.43	< 0.001	1550	1529.3–1570.7	< 0.001	1306	1298.02–1313.98	< 0.001	445	441.57–448.43	< 0.001	532	521.88–542.12	< 0.001
Year 2019	15	−92.78–122.78	0.785	26	−2.99–54.99	0.079	−46	−57.11–−34.89	< 0.001	14	9.19–18.81	< 0.001	71	56.26–85.74	< 0.001
Year 2020	67	−40.89–174.89	0.224	15	−12.78–42.78	0.29	25	14.09–35.91	< 0.001	114	107.12–120.88	< 0.001	134	117.43–150.57	< 0.001
Year 2021	288	171.14–404.86	< 0.001	76	48.97–103.03	< 0.001	106	95.77–116.23	< 0.001	82	76.17–87.83	< 0.001	163	145.58–180.42	< 0.001

*Hospital*															
LT1	1164	1092.17–1235.83	< 0.001	1134	1115.58–1152.42	< 0.001	934	926.25–941.75	< 0.001	561	555.38–566.62	< 0.001	552	540.84–563.16	< 0.001
HT2	406	302.72–509.28	< 0.001	617	591.97–642.03	< 0.001	426	413.23–438.77	< 0.001	68	59.42–76.58	< 0.001	163	145.83–180.17	< 0.001
LT3	−141	−249.11–−32.89	0.011	164	135.47–192.53	< 0.001	91	78.8–103.2	< 0.001	−132	−139.82–−124.18	< 0.001	102	85.59–118.41	< 0.001
HT3	−61	−222.33–100.33	0.459	520	474.55–565.45	< 0.001	614	601.97–626.03	< 0.001	−185	−191.71–−178.29	< 0.001	−63	−85.15–−40.85	< 0.001
LT2	−67	−255.38–121.38	0.486	498	469.22–526.78	< 0.001	381	367.52–394.48	< 0.001	−228	−234.49–−221.51	< 0.001	−175	−194.82–−155.18	< 0.001
LT4	−333	−628.53–−37.47	0.027	−19	−52.9–14.9	0.272	−140	−152.21–−127.79	< 0.001	−176	−183.26–−168.74	< 0.001	−215	−234.41–−195.59	< 0.001
HT1	171	47.11–294.89	0.007	1096	1034.5–1157.5	< 0.001	823	809.43–836.57	< 0.001	131	120.14–141.86	< 0.001	296	274.92–317.08	< 0.001
LT5	−190	−777.86–397.86	0.526	197	154.63–239.37	< 0.001	285	267.73–302.27	< 0.001	−206	−213.04–−198.96	< 0.001	−238	−250.79–−225.21	< 0.001

## Data Availability

All relevant data are included within the manuscript. Complete dataset cannot be shared by the investigator because all data were collected from the electronic health records system with the authorization of the Catalan Institute of Health.
